# Malignant Adenoma of the Kidney

**Published:** 1902-11-08

**Authors:** 


### MALIGNANT ADENOMA OF THE KIDNEY.

A remarkable case, and one which must serve to brighten the gloomy prognosis which hangs over all cases of tumour of the kidney in children, was shown to the last meeting of the Clinical Society by Dr. J. D. Malcolm. The patient, from whom in November, 1892, he had removed a malignant adenoma of the right kidney, he now exhibited. She was now 11 years and 10 months old, and enjoyed good health, although she was rather paler than her brothers and sisters. The kidney with the tumour attached, which he had removed ten years ago, was preserved in the Museum of the Royal College of Surgeons. All the capsule of the kidney and some enlarged glands on the renal vessels were removed. Mr. Targett had examined the tumour and described it as "malignant adenoma." Only two cases in which tumours of the kidney had been removed from children who had survived the operation for two years, were known to Mr. Malcolm. Both of these had been operated on by Dr. Abbé, of New York, but in one of these, three and a half years after the operation, sarcoma developed in the opposite kidney, and life was rapidly destroyed. The President, in congratulating Mr. Malcolm on the case, said that he could not recall a single instance in which malignant disease of the kidney in a child had been operated on with permanent success.

